# Supplementary material for: Impact of axial length correction in high intraocular pressure eyes on intraocular lens power calculation
Source: PLoS One. 2026 May 20;21(5):e0349117. doi: 10.1371/journal.pone.0349117 (PMC13189341; doi:10.1371/journal.pone.0349117)
Supplement: S1 Table — We compared ACD and LT between the high IOP group in the current study and 57 eyes from 57 patients with normal IOP who underwent cataract surgery alone, matching for age, axial length (AL), and the model of intraocular lens (AN6KA, KOWA). IOP = Intraocular Pressure; ACD = Anterior Chamber Depth; LT = Lens Thickness. * Wilcoxon signed-rank test. (DOCX) [file pone.0349117.s001.docx]

|  | Normal IOP group | High IOP group | *P* value* |
| --- | --- | --- | --- |
| ACD (mm) | 3.02 ± 0.38 | 2.21 ± 0.35 | <.000 |
| LT (mm) | 4.62 ± 0.45 | 5.19 ± 0.29 | <.000 |
